# Tachycardia-Induced Cardiomyopathy Secondary to Iatrogenic Chronic Hyperthyroidism

**DOI:** 10.7759/cureus.97441

**Published:** 2025-11-21

**Authors:** Yan Jack Chung, Avni Kant, Susie Lewis

**Affiliations:** 1 Cardiology, Salisbury District Hospital, Salisbury, GBR; 2 Internal Medicine, Salisbury District Hospital, Salisbury, GBR

**Keywords:** decompensated heart failure, heart failure with reduced ejection fraction, iatrogenic cardiomyopathy, liothyronine, tachycardia-induced cardiomyopathy

## Abstract

This case describes a middle-aged woman found to have tachycardia-induced cardiomyopathy secondary to chronic liothyronine overuse. Despite a non-specific presenting complaint, she was found clinically to have heart failure and evidence of fluid overload. ECG showed that she was in atrial flutter. A chest X-ray showed marked cardiomegaly and bilateral pleural effusions. Imaging of the abdomen showed a dilated inferior vena cava and evidence of systemic congestion. Trans-thoracic echocardiography showed a left ventricular ejection fraction of under 30%. Thyroid function tests showed a severely suppressed endogenous thyroid function.

The patient was admitted under cardiology and given diuresis, including a stay in intensive care following an episode of acute hypotension. After stabilisation and discharge, her endogenous thyroid function and left ventricular systolic function are slowly recovering, and she reverted spontaneously to sinus rhythm.

## Introduction

Heart failure is an increasingly prevalent healthcare issue, associated with high mortality and symptom burden [1]. It is important to identify any reversible causes, as treatment will impact the patient’s symptoms and future health. Tachycardia-induced cardiomyopathy (T-CMP) is a reversible form of left-ventricular (LV) dysfunction that can arise from any tachyarrhythmia. Although the loss of cardiac function is reversible, there is no guarantee of complete recovery.

The increased risk of arrhythmias in a chronic hyperthyroid state can lead to T-CMP. It is well known that hyperthyroidism increases heart rate and myocardial contractility [2]. In response to stimulation by thyroid-stimulating hormone (TSH), the thyroid produces triiodothyronine (T3) and thyroxine (T4) that are carried through the circulation to target tissues and organs. T4 is largely inactive and is converted into T3 in the liver, kidneys, and skeletal muscle by the enzyme deiodinase. The active form of thyroid hormone is T3, which in drug form is known as liothyronine. The effects of chronic high-dose liothyronine intake are not widely documented; however, hyperthyroidism can cause heart failure, palpitations, atrial fibrillation, and exercise intolerance [3]. This is a case of T-CMP secondary to atrial flutter due to iatrogenic hyperthyroidism.

## Case presentation

A woman aged 66 years presented to the hospital with a sudden onset of upper abdominal pain and a four-day history of fatigue, loss of appetite, nausea, legs giving way, and breathlessness. She was unable to climb up stairs (NYHA class III). There was a family history of myocardial infarction that had affected both her brothers. On examination, there was guarding in the right upper quadrant of her abdomen. She was clinically fluid overloaded with a raised jugular venous pressure and lower limb oedema to her mid-shins. She was admitted under the surgical team, who investigated her with abdominal ultrasound and CT abdomen and pelvis (CTAP) with contrast, and found no acute surgical cause for her symptoms. She was referred to cardiology for treatment of heart failure.

When she was reviewed by cardiology, it was noted that she had ordered liothyronine (T3 hormone) online and had been taking 200 micrograms daily (the usual recommended dose is 10 to 20 micrograms per day). About 10 years before, she had been under the care of an endocrinologist who had told her that she was unable to metabolise T4 to T3. In recent years, she had requested her own blood tests, which consistently showed low T4 and TSH levels, and adjusted the liothyronine dose depending on her level of fatigue and on serum levels of T3, which were usually low. When told that we would usually aim for a normal TSH, she said that she had previously been advised by her endocrinologist (now deceased) that negligible TSH is acceptable.

Cardiac investigations

**Figure 1:**
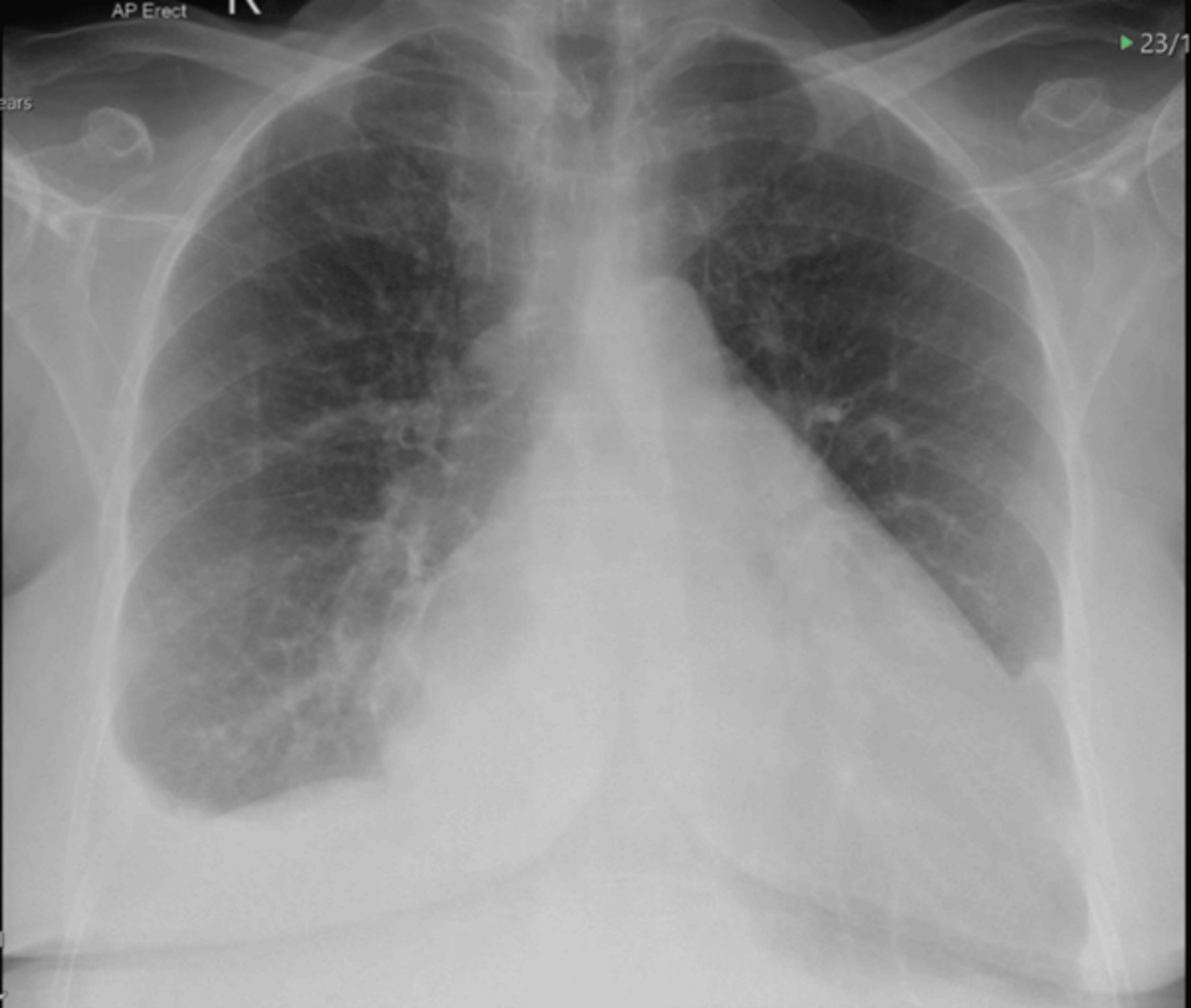
Anteroposterior chest radiograph showing cardiomegaly and bilateral pleural effusions.

The diagnosis of heart failure was supported by the investigations initiated by the surgical team for her right upper quadrant pain. A chest X-ray revealed cardiomegaly and bilateral pleural effusions (Figure 1). CTAP with contrast showed a prominent inferior vena cava and hepatic veins. N-terminal pro-B-type natriuretic peptide was elevated at 2686 ng/L. All these findings were consistent with heart failure.

**Figure 2:**
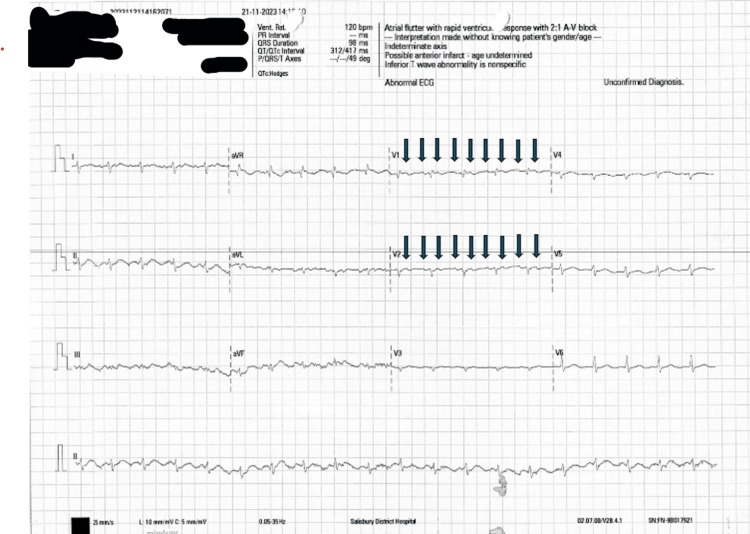
ECG showing atrial flutter with 2:1 block, with flutter waves most visible in leads V1 and V2. ECG, electrocardiogram.

The echocardiogram showed severely impaired left ventricular systolic function, with a left ventricular ejection fraction (LVEF) of <30%. Right ventricular systolic function was also impaired with biatrial dilatation. Functional moderate mitral and moderate-to-severe tricuspid regurgitation were observed. The ECG showed atrial flutter with 2:1 block, with an atrial rate of around 240 and a ventricular rate of 120 (Figure 2). A repeat focal echocardiogram was performed five days later when the patient developed an episode of marked hypotension. This showed similar findings. LVEF remained severely reduced 19 days after admission, before discharge, at 10%-15%.

Endocrine investigations

Thyroid function tests were performed the day after her CT scan and showed low TSH at <0.01 mU/L and low free T4 of <0.5 pmol/L, demonstrating suppressed endogenous thyroid function.

Diagnosis

She was diagnosed with heart failure with reduced ejection fraction (HFrEF). The cause of heart failure was cardiomyopathy, which was thought to be tachycardia-mediated, due to rapid atrial flutter secondary to chronic overtreatment with liothyronine.

Inpatient management

She was treated with intravenous furosemide for fluid overload. Because the diagnosis was HFrEF, she was commenced on medication to optimise cardiac function, following National Institute for Health and Care Excellence guidelines for HFrEF [4]. This included ramipril, bisoprolol, dapagliflozin, and spironolactone. She was also started on apixaban as anticoagulation due to the diagnosis of atrial flutter, with a plan for outpatient cardioversion.

High-dose diuretics resulted in hypotension and required support with vasopressors. She was transferred to the intensive care unit and treated with metaraminol, to which she responded well. She received intravenous fluids, and furosemide and Bisoprolol were withheld. She was given a short course of milrinone.

Once weaned off metaraminol and milrinone, she was transferred to the cardiac ward and cautiously restarted on intravenous diuretics. She had strict fluid balance monitoring and a minimum of 1.5 L oral fluid intake to maintain right-sided cardiac pressures. A week later, she was approaching euvolaemia and changed to oral furosemide.

Outcome and follow-up

Under the advice of endocrinology, her liothyronine dose was reduced from 200 mcg daily to 10 mcg three times each day, and she was discharged on this dose. At initial follow-up, thyroid function had started to recover. TSH was 0.2 mU/L, and six months later, it rose to 0.31 mU/L, which is within the normal range (Table 1). She was maintained on 10 mcg of liothyronine three times a day.

**Table 1:** Thyroid function tests over time showing suppressed endogenous thyroid function and gradual recovery. fT4, free thyroxine; TSH, thyroid-stimulating hormone.

Date	TSH (normal: 0.27-4.2 mU/L)	fT4 (normal: 11-22 pmol/L)
24/11/2023	<0.1	<0.5
07/12/2023	<0.1	0.7
08/12/2023	<0.1	<0.5
10/12/2023	<0.1	0.8
11/12/2023	<0.1	0.6
12/12/2023	<0.1	0.6
29/12/2023	0.23	1
08/02/2024	0.21	1.5
13/02/2024	0.2	1.4
20/05/2024	0.25	1.4
05/11/2024	0.42	1.2
10/07/2025	0.34	1.6

Six weeks after admission to the hospital, a repeat echocardiogram showed an improvement in LVEF to 25%-30%, with further improvement six months later to 50%-54%. At six months, she had reverted to sinus rhythm spontaneously, so she did not require outpatient cardioversion. About 12 months later, she had remained in sinus rhythm, and the echocardiogram showed a similar ejection fraction.

## Discussion

There are several published case studies on T-CMP that demonstrate a similar scenario of a patient with longstanding tachycardia presenting with evidence of heart failure and poor ejection fraction, whose LVEF recovered after the tachycardia was treated [5-8]. This case demonstrates a recovery of LVEF in a longer timeframe than in these studies. This could be due to prolonged atrial flutter, paired with very severely impaired systolic function, resulting in a slower recovery. There is a risk of recurrence; a study showed that after recovery from T-CMP, patients with recurrence of tachyarrhythmia had a more rapid deterioration in LV systolic function compared to previous episodes [9].

A key element of managing T-CMP is correction of the causative tachyarrhythmia. Radiofrequency catheter ablation is preferred as first-line therapy for certain tachyarrhythmias. It has a high success rate for treating atrial flutter, atrial tachycardia, atrioventricular nodal re-entrant tachycardia, and atrioventricular reciprocating tachycardia. In T-CMP caused by atrial fibrillation, rate-control is the favoured strategy because there is a high rate of recurrence after cardioversion [9]. It is also advised to treat HFrEF in T-CMP with the usual medications such as beta-blockers, angiotensin-converting enzyme inhibitors or angiotensin receptor blockers, mineralocorticoid receptor antagonists, and sodium-glucose cotransporter-2 inhibitors [9].

It has been shown that urinary T3 is not an accurate diagnostic test for hypothyroidism [10]. Hence, multiple free T3 assays showed levels below the normal range despite the patient taking a supraphysiological dose of liothyronine. It is uncommon to see liothyronine used as treatment for hypothyroidism. This patient underwent some investigations under the advice of a private endocrinologist. She was originally diagnosed with hypothyroidism after a finding of low urinary T3, and started on natural desiccated thyroid extract, but was changed to liothyronine in 2011. This was continued for 12 years despite low serum TSH and normal free T3. It is known that liothyronine is more rapidly metabolised than levothyroxine and has a more rapid effect; thus, liothyronine may be used in severely hypothyroid states where conversion to T3 is decreased, whereas levothyroxine is used as maintenance treatment in hypothyroidism [11].

There is little literature exploring the efficacy and side effects of using liothyronine monotherapy instead of levothyroxine for the management of hypothyroidism. A large study found an increased risk of stroke and heart failure in patients taking liothyronine compared to those taking levothyroxine, with a greater difference in those on liothyronine for more than one year [12]. Jonklaas et al. found that with a once-daily dosing regimen of liothyronine, serum concentrations of T3 show a significant degree of variation [13] and alongside knowledge that the physiological effects of liothyronine are very transient [14], this explains why levothyroxine is given once daily, whilst liothyronine is normally given in two to three divided doses over the course of a day. Given the supraphysiological doses of liothyronine taken by the patient in our case study, it was no surprise she developed tachycardia, which is known to precipitate heart failure [15].

## Conclusions

This case emphasises the importance of checking thyroid function in patients with heart failure, especially where there is tachycardia. The patient’s private use of liothyronine was the cause of atrial flutter and heart failure. There is much more literature about the relationship between hyperthyroidism and atrial fibrillation, rather than atrial flutter, and atrial fibrillation is the rhythm classically associated with hyperthyroidism.
